# Translation, Adaptation, and Psychometric Validation of the Spanish Version of the Attitudes towards Research and Development within Nursing Questionnaire

**DOI:** 10.3390/ijerph19084623

**Published:** 2022-04-12

**Authors:** Silvia Gros Navés, Olga Canet-Vélez, Williams Contreras-Higuera, Judith Garcia-Expósito, Jordi Torralbas-Ortega, Judith Roca

**Affiliations:** 1 Department of Nursing and Physiotherapy, Faculty of Nursing and Physiotherapy, University of Lleida, 25198 Lleida, Spain; silvia.gros@udl.cat (S.G.N.); judithga1127@gmail.com (J.G.-E.); 2 Faculty of Health Sciences Blanquerna, University Ramon Llull, 08025 Barcelona, Spain; olgacv@blanquerna.url.edu; 3 Department of Methods of Research and Diagnosis in Education, Faculty of Education, University of Barcelona, 08035 Barcelona, Spain; williams.contreras@gmail.com; 4 Parc Taulí Hospital, Nursing Research Group (@GRItauli) of Research and Innovation Institute Parc Taulí (I3PT), 08208 Sabadell, Spain; jorditorralbas@gmail.com; 5 Health Care Research Group (GRECS), Biomedical Research Institute of Lleida, 25198 Lleida, Spain

**Keywords:** nursing, research, research competence, professional competence

## Abstract

The promotion of research competence is essential for the development of the nursing profession and discipline. The aim of this study was to translate into Spanish, adapt, and validate an instrument measuring nurses’ attitudes towards nursing research and development. A quantitative, cross-sectional, analytical design was used for the cross-cultural adaptation and cultural validation of the instrument. A total of 367 participants were selected using intentional sampling. A process of translation, back-translation, expert consultation, and pilot testing was followed. Subsequently, reliability and statistical validity were assessed, a new factor structure was proposed, and means were compared to assess the power to discriminate between factors by groups of participants. The results showed internal consistency tests with a Cronbach’s alpha of 0.913. Confirmatory factor analysis of the comparative fit index (CFI = 0.549) and Tucker–Lewis index (TLI = 0.491) indicate that the factors did not match the original clustering model. The new factor structure consisted of seven factors. Between-group comparisons revealed statistically significant differences. In conclusion, the instrument exhibits high levels of statistical reliability and validity compared to the original instrument. The new factorial proposal is consistent, but further research is needed to verify its replicability in other contexts.

## 1. Introduction

Nursing, as a scientific discipline, must build its knowledge base on high quality research [1], so it is essential to develop research competence among nurses. Research competence may be defined as the ability to conduct sustainable collaborative research activities in a specific setting [1]. Collaborative research including research involving clinicians and academics or experienced and junior researchers increases knowledge and improves skills in a safer, more motivating environment [2]. Individuals feel more valued and feel that their contributions are more recognised when part of a research team. In addition, high-quality research in health requires a joint effort from researchers, and it is therefore essential that nurses join or form research teams as a fundamental step toward the visibility and consolidation of their work [3]. Problems arising in the health care setting (i.e., clinical scenarios) should be addressed in different ways, at different times, and by different professionals, but this is not always the reality of the situation [4]. To this end, dynamic interventions must be implemented and funded at various levels or foci in order to truly bring about social changes favouring nursing [5]. Specific strategies should be based on building infrastructure, fostering a culture and environment conducive to research, and facilitating training and collaboration [6]. Training is key to generating further knowledge and skills as well as in identifying factors that could lead to a lack of support for nurses in a particular setting and to prevent their realisation [7].

The situation of nursing research in Spain, which is the framework for this study, is characterised above all by the structuring of qualifications within the European Higher Education Area (the 1999 Bologna Declaration), which made it possible to upgrade from a diploma in nursing to a degree in nursing, regulate masters’ degrees in nursing, and obtain a doctorate in nursing [8]. Access to specific doctoral studies in nursing facilitates the conduct of research within a nursing-, care-based conceptual framework [9]. The link between having doctoral programmes available and producing further research is more than expected [3].

In the last decade, there has been a growth in academia and science, and nursing has been established as a science capable of producing its own knowledge and research [8]. A number of recent studies paint an encouraging picture, in particular, a study carried out in a Spanish health care facility in 2020 [10], which reported that participants viewed research as a necessary activity (88.8%) that helped to develop the profession (98.9%), although it required a large investment in terms of time and effort (94.9%). This positive perception towards research has also been reported among senior nursing students [11], who also exhibited a good level of self-perceived research competence [12,13]. Another encouraging aspect is the recognition by the Spanish Royal Decree 822/2021 in 2021 of the specific area of knowledge of nursing as separate from medicine and the generic group of health sciences [14].

A literature review [15] revealed the existence of a variety of instruments to assess research-associated competences, but there is a lack of studies on valid, reliable instruments. Most of the available instruments are written in English and therefore need to be translated and validated [16]. The instrument on nurses’ attitudes and motivation regarding research by Cepeda et al. (2009) [17], in Spanish, had an overall Cronbach’s alpha of 0.86, but its validation was carried out using a very small sample of nurses (*n* = 77), which may lead to methodological limitations. As a result, translating, adapting, and validating another instrument was considered. It was decided to select an instrument that addressed research competence in general and not only evidence-based practice [18,19] and that was international in nature, in order to be able to compare data and make data more widely available. In addition, the instrument that is the topic of the present study had been used in different settings, and not only with professionals, but also with nursing students [20,21,22]. 

The primary objective of this study was therefore to translate into Spanish, adapt, and validate an instrument in English on attitudes towards research and development in nursing. Secondary objectives included describing any differences with the original questionnaire, presenting the new factor groupings, and assessing the discriminating power of the proposed factors by comparing groups of participants. 

## 2. Materials and Methods

### 2.1. Design

The study used a cross-sectional analytical design for the cross-cultural adaptation, validation, and reliability assessment of an instrument on attitudes towards research and development in nursing. The translation and adaptation process requires verifying the metric properties of the two cultures involved (i.e., Swedish and Spanish) [23].

### 2.2. Participants

A convenience sample of 367 participants was selected from a reference population of nurses and nursing students.The study was carried out at the Igualada Campus of the Faculty of Nursing and Physiotherapy of the University of Lleida and at the Igualada University Hospital. As the necessary sample was not obtained, nurses from other hospital facilities affiliated to the University of Lleida were included in the study. The sample size was based on a person–item ratio of at least 10 subjects per item in the instrument for general psychometric approaches and 300–500 subjects per item for confirmatory factor analyses or power analyses [16]. The inclusion criterion was to either be a registered nurse or be a student in the final academic year of their nursing degree. Students in their final year were eligible as it was considered that they would possess greater knowledge of nursing compared to other students of nursing and would be prepared to enter the workforce. The only exclusion criterion applied was being a retired nurse.

### 2.3. Measuring Instruments 

The use of the original version of the questionnaire was authorised via email by the original authors in October 2018, who provided the 2002 English version (II) of the questionnaire. The overall Cronbach’s alpha value was 0.94, indicating a good level of internal consistency for all 34 items in the questionnaire [20]. Responses to the statements used the original 5-choice Likert format: (1) do not agree at all; (2) agree to a very little extent; (3) agree to a certain extent; (4) agree to a great extent; and (5) agree to a very great extent. The factor dimensions of the original instrument, the number of statements associated with them, and their Cronbach’s alpha values are presented below [20,21]:

- Factor 1 ‘Research language’ describes the language used in scientific and nursing research (two statements, Cronbach’s alpha: 0.78).
- Factor 2 ‘Need for research knowledge’ describes nursing research and the nursing researcher as necessary for nursing practice (five statements, Cronbach’s alpha: 0.80).
- Factor 3 ‘Participation’ describes the nurses and the extent to which they participate in nursing research in their daily work (six statements, Cronbach’s alpha: 0.84).
- Factor 4 ‘The Profession’ describes the nursing profession and its inherent professional skills as either vocationally orientated or academically orientated (five statements, Cronbach’s alpha: 0.77).
- Factor 5 ‘Meaningfulness’ describes the nurses’ interest in and use of nursing research in the nursing area (five statements, Cronbach’s alpha: 0.75).
- Factor 6 ‘Study literature’ describes the need to read articles and research reports about nursing and to keep up to date in other ways (six statements, Cronbach’s alpha: 0.78).
- Factor 7 ‘Development—Resources’ describes the nursing profession as including the testing of new ideas that are based on experience and science, as well as the importance of human resources at the workplace (five statements, Cronbach’s alpha: 0.60).

The form used in this study consisted of three parts: (a) basic sociodemographic data (age and sex); (b) questions, adapted to the Spanish context, on training (initial or advanced), work experience (years and location), and research experience (principal investigator or collaborator, types of tasks performed, and publications); and (c) statements on attitudes towards nursing research and development.

### 2.4. Procedure 

Version II of the instrument published by Björkström and Hamrin (2001) [20] was translated from English into Spanish and adapted to the Spanish context. The phases of the translation process as described by the World Health Organisation [24] as well as the recommendations outlined by Sousa and Rojjanasrirat (2011) [16] and Kalfoss (2019) [25] were followed. Two translators (one expert in health terminology and one expert in linguistics) participated independently in the translation and back-translation process. It was requested that a panel of experts, consisting of three participants, assess the relevance, clarity, and comprehensibility of the instrument. Subsequently, the instrument was pilot-tested for comprehensibility and duration with 25 professionals.

The translation and proofreading phase revealed that statement 14 (‘The nursing education programme is also research-based’), which appeared in version II, was not present in the studies published in 2001 and 2003 and was therefore removed from the Spanish version. Other studies [22] have already adapted the scale in a similar way.

The wording of a number of translated statements (1, 3, 7, 17, 22, 23, 31, and 34) was modified by the expert panel to improve their comprehensibility. The experts, together with the researchers, decided to create a section in the questionnaire with questions on training, work experience, and research experience adapted to the Spanish context on the basis of the original proposal (version II translated into English and provided by the authors). With regard to the pilot test, 25 professionals participated and completed the test in a mean time of 24.45 min (SD = 5.2 min). They did not suggest any changes to the wording of the statements.

### 2.5. Data Collection

Data collection took place between May 2019 and February 2020. The questionnaire was administered on paper by researchers and collaborators from the health care facilities. Completion time ranged from 20 to 35 min.

### 2.6. Ethical Considerations

The study was approved by the CAERFIF Research Committee for the Faculty of Nursing and Physiotherapy of the University of Lleida and by the Ethics Committee of the Igualada University Hospital. Authorisation was obtained from all health care facilities involved.

All participating nurses and students gave their informed consent. All data were processed confidentially and anonymously.

### 2.7. Data Analysis

A descriptive analysis of the study variables was carried out. Cronbach’s alpha was used to assess reliability, and confirmatory factor analyses (CFA) in relation to the original English version were performed for factor validity. As no concordance was found between the factor structure of the Spanish version and that of the original instrument, an analysis was carried out to determine the new factor structure using the same extraction (maximum likelihood) and rotation (Oblimin) methods for seven factors. Finally, in order to assess the discriminating power of these new factors, means of sociodemographic variables (e.g., training) were compared between groups.

Data were analysed using IBM SPSS Statistics 24 (IBM Corp., Armonk, NY, USA) and JASP 0.14.1 software (JASP Team, Amsterdam, The Netherlands). The statistical significance threshold was set at *p* < 0.05 for all analyses.

## 3. Results

A total of 367 nurses and students participated, 85% of whom were female (*n* = 306). Ages ranged between 21 and 63 years (x = 34.19, SD = 11.29); 83.9% were working registered nurses and 16.1% were nursing students in their final academic year. Nurses’ advanced training was divided into doctorate, master’s degree, postgraduate diploma, and continuing education. Their years of work experience were distributed as follows: 0–5 years (32.8%), 6–10 years (11.3%), 11–15 years (14.6%), 16–20 years (12.9%), and >20 years (28.5%). Their expertise was focused mostly on hospital care activities (55.9%). It is important to note that almost half of the respondents (*n* = 149) had participated in research projects at some point in time. Their specific contributions focused on data collection (36%), participating as research collaborators (26.7%), searching for information (21%), and writing reports (13.6%). Only 12.5% had experience as principal investigators and 14.7% had experience in article publication. Naturally, the group of students did not possess advanced training or work experience, and reported that they had not participated in any external research, their only research experience being the preparation of their final degree project (FDP) (Table 1).

With regard to the cultural validation of the instrument, the reliability of the overall instrument was calculated using the negative items previously reversed. The set of items had a high level of internal consistency (α = 0.913). In item deletion tests, there were no items whose deletion significantly altered the Cronbach’s alpha value.

A confirmatory factor analysis (CFA) was performed on the results and the factors were grouped as proposed by Björkström and Hamrin (2001) [20]. The analysis revealed a low level of concordance with the hypothesised model. The comparative fit index (CFI = 0.549) and Tucker–Lewis index (TLI = 0.491) values showed that the factors did not match the theoretical model for clustering (values above 0.9 are accepted), and consequently, the same results as in the original model could not be attained. 

As it was not possible to reconstruct the factor components of the original study, a new factor analysis was performed by replicating Björkström’s original procedure using the maximum likelihood extraction method and Oblimin rotation (delta, 0). The number of factors to be extracted was set at seven in order to emulate the procedures outlined by Björkström and Hamrin (2001) [20] and the results were then analysed. Correlation coefficients have been previously verified using the anti-image correlation matrix. Item 28 (‘Proficiency in nursing is primarily attained through long practical experience’) obtained the lowest correlation coefficient (r = 0.718) and was removed because it affected the internal consistency of two factors.

A Kaiser–Meyer Olkin (KMO) value of 0.916 and a Bartlett’s test of sphericity value of gl = 528, *p* = 0.000 were obtained. The resulting seven factors explained 53.92% of the cumulative variance. Table 2 presents the seven factors along with their definitions, the number of statements, and the resulting Cronbach’s alpha values per factor.

Table 3 presents the seven factors along with their statements ordered by factor loading. Two statements (2 and 8) were moved from F2 to F5 for conceptual consistency reasons (i.e., the logic of the propositions used, making daily professional practice more coherent) after verifying that the Cronbach’s alpha was not affected. 

In order to assess the discriminatory power of the new proposed factors, a between-group comparison test (basic vocational training) was carried out. The analysis of variance (ANOVA) showed a number of differences between the three main groups. Differences between groups of participants (nurses with a Diploma of Higher Education in Nursing, nurses with a Degree in Nursing, and nursing students) could be observed. Nursing students scored the highest on all seven dimensions, especially on evidence-based practice ($\bar{x}$ = 4.47), assessment of nursing research and development of the nursing discipline ($\bar{x}$ = 4.49), and assessment of nursing research and development as applied in daily professional practice ($\bar{x}$ = 4.37). In stark contrast, nurses with a Diploma of Higher Education in Nursing scored the lowest on all factors, the lowest scoring factors being language of research ($\bar{x}$ = 3.65) and the development of professional and research skills ($\bar{x}$ = 3.76). However, nurses with a Degree in Nursing had intermediate scores on all factors. Finally, ANOVA revealed that the factors had a good structure to be able to discriminate between groups. Factors F1, F5, and F7 were found to have statistically significant differences between all three groups: nurses with diplomas, graduates, and students (Table 4). 

## 4. Discussion

The aim of this study was to translate into Spanish the instrument “Attitudes towards research and development within nursing” and validate the translated version. The results showed that the instrument had a very high level of reliability (α = 0.913); however, its statistical validity failed to match the results of the factors in the original version by Björkström and Hamrin (2001) [20] using a confirmatory factor analysis (CFA). This factorial difference was also reported by Toraman et al. (2017) for the Turkish version [22]. This suggests that some components of the original version are unstable when the scale undergoes modifications. It should also be noted that Frasure (2008) [15] conducted a systematic review of 14 instruments measuring nurses’ attitudes towards the use of nursing research including this instrument. This author pointed out that if the theoretical framework of an instrument is unclear, then the items matching its component factors are likely to be confusing. Björkström and Hamrin (2001) [20] did not report on the type of validity or the specific theoretical model used for the instrument.

The newly proposed factor structure, consisting of seven factors, resembled the original version [20]. These factors account for approximately 50% of the factor loadings, with a KMO value of 0.916, suggesting that the grouped variables were strongly related and therefore correctly grouped. Subsequently, their definitions emerged from the authors’ analytical work on the literature. F1 (Linkages between academia and the workplace) shows the relationship between practitioners and academics as the main actors in the development of high-quality research [26] as well as the need for continuous collaboration. F2 (Assessment of nursing research and development of the nursing discipline) refers to an established and stable nursing discipline that seeks the constant evolution of nursing knowledge, new areas of knowledge, and room for the development of competencies [27] and nursing theory [28]. F3 (Language of research) entails the need for critical reading as an essential component of understanding research for professionals and students alike [29]. F4 (Development of professional and research skills) includes not only aspects of research methodology but also (foreign) language comprehension (e.g., English, due to the vast scientific production in this language) and the socialisation or dissemination of research results [30]. With regard to F5 (Assessment of nursing research and development as applied in daily professional practice), a study by Hopia and Heikkilä (2020) stressed the importance of and need for further clinical research from a nursing perspective in order to improve disease management and patient health outcomes at the individual and organisational level. F6 (Willingness to promote the development of nursing) refers to the desire to integrate nursing research into the nursing profession and to socialise nursing research through scientific events [30]. Finally, F7 (Evidence-based practice) addresses the need to develop empirical and scientific knowledge based on the introduction of changes founded on the integration of scientific information, which is the essence of evidence-based practice (EBP). Taking a holistic approach, EBP could be defined as the use of research when evidence (research findings) is implemented [31]. However, this evidence must be integrated together with the philosophical foundations shaping the discipline at hand [32].

Other aspects such as the passing of time and biases associated with the culture of nursing research may also explain the factorial variability observed in the components of the instrument. Nursing research in Spain started late, up to two decades later than in other parts of the world. While in Scandinavia there was already a group of doctors in nursing in 1998, doctoral programmes in nursing in Spain only started in 2005 as a prerequisite for joining the European Convergence Process under the common credit system (ECTS) [33].

In addition, nursing research findings should be of great interest to nurses, otherwise they might be perceived as too abstract and of little use for day-to-day nursing practice [20]. Initiatives such as ‘Shaping Better Practice Through Research: A Practitioner Framework’ [34] and ‘Leadership Mentoring in Nursing Research’ [35] are therefore highly relevant in helping to develop research frameworks and in supporting nursing professionals and students to achieve optimal practice and outcomes. Research competence must generate and validate knowledge to solve practice-related issues and improve the quality of care and the quality of life of the individuals involved [30]. Knowledge transfer is extremely important for a profession to become established and ensure that patients receive the highest quality, up-to-date, evidence-based health care [36]. This requires instruments that correctly assess research competence. Thus, the instrument presented in this study proposes a multifaceted approach to nurses’ attitudes towards nursing research and development through the seven factors it encompasses, facilitating an in-depth analysis of research competence in any care setting and at different points in time (e.g., initial assessment of a group, performance, or other). This approach also uses clear language, with simple, efficient wording, and easy-to-interpret results.

Finally, the newly proposed factor structure was shown to discriminate between groups, and its constituent items were consistent with each other. The group analysis showed differences between groups, in favour of students in a number of factors. Following the introduction of the nursing research competence and evidence-based nursing practice, a nursing degree has become a driving force in the stimulation and motivation of students [37] and, after their transition to the world of work, of the entire scientific community. Further research is needed on the individual, organisational, and environmental factors that explain why nurses with a Diploma of Higher Education in Nursing or a Degree in Nursing rate research competence poorly. This would shed light on the actual assessment of the impact and development of the research competence in nursing practice [38]. Programmes should be put in place to boost nurse research leadership in clinical work while avoiding tensions between practice, research, and teaching settings [39]. However, it is certainly clear that ongoing collaboration between professionals, academics, and stakeholders is needed to properly conduct joint research and set priorities to advance the nursing discipline and the nursing profession [28].

## 5. Limitations 

The main limitation of the study is its design, which used a non-randomised sample and unbalanced, diverse groups of nurses and nursing students together. Consequently, this study should be considered to be exploratory research. In addition, the need to establish theory-based groups becomes evident, as the internal consistency of the dimensions becomes affected, with only marginally acceptable values (α < 0.7). Therefore, this limitation can be said to have been inherited from the original study.

Furthermore, being a self-reporting instrument, this study may also suffer from social desirability biases [18]. Finally, its suitability for use with nursing students should also be reconsidered in the future, as this instrument was initially designed for use with professionals [40].

## 6. Conclusions

The Spanish version of the instrument ‘Attitudes towards research and development within nursing’ displayed high levels of statistical reliability and validity compared to the original version. However, this cannot be confirmed, due to among other aspects, the fact that the original instrument is lacking in construct validity and the fact that there are differences in time and culture between the original version and the translated version. Nevertheless, the new proposed factors are consistent with each other. Further studies are needed to verify the replicability of the proposed factors beyond the study sample and the context described [41]. The Spanish instrument includes 33 items, as two items (14, 28) were removed from the original instrument.

Another relevant aspect is the contextual validation carried out in this study. The nursing environment is constantly evolving, so it is essential to be able to measure the development needs of nursing research in order to implement more adapted interventions that truly link the nursing profession and nursing research.

## Figures and Tables

**Table 1:** Characteristics of the sample: number (*n*) and proportion (%).

Variables		*n*	%
Age *		34.1	11.29
Sex	Male	54	15.0
	Female	306	85.0
Initial training	Diploma of Higher Education in Nursing	198	53.9
	Bachelor’s Degree in Nursing	110	30
	Student	59	16.1
Advanced training	Doctorate	7	2.3
	Master’s degree	157	51
	Postgraduate diploma	91	29.5
	Continued professional development courses	53	17.2
Work experience	Hospital care	205	55.9
	Specialised units	175	47.7
	Primary care/Community care	60	16.3
	Nursing homes and health and social care	56	15.3
	Teaching	56	15.3
	Management	31	8.4
Research experience	Principal investigator	46	12.5
	Research collaborator	98	26.7
	Data collection	132	36.0
	Information search	77	21.0
	Preparation of reports	50	13.6
	Publications in journals	54	14.7

* Mean and standard deviation (SD).

**Table 2:** Factor definitions.

Factors	Definition	No. of Statements	Cronbach’s Alpha
F1. Linkages between academia and the workplace	Specifies the need for networking with faculty and students to encourage nursing development at the clinical level	2	0.614
F2. Assessment of nursing research and development of the nursing discipline	Details the importance nurses attach to research and curriculum development at the disciplinary level	8 (−2)	0.731
F3. Language of research	Describes the scientific language used in articles, necessary for understanding and interpreting applied methodologies and research findings	2	0.748
F4. Development of professional and research skills	Specifies the strategies for research skills acquisition and professional development	6	0.706
F5. Assessment of nursing research and development as applied in daily professional practice	Describes the importance that nurses place on research and professional development in their day-to-day professional work	7 (+2)	0.755
F6. Willingness to promote the development of nursing	Describes a positive attitude towards the inclusion of new knowledge and research to improve professional practice	4	0.648
F7. Evidence-based practice	Involves the integration of scientific information and the introduction of changes based on empirical and scientific knowledge	4	0.742

**Table 3:** Pattern Matrix. Grouped factors and statements.

Factors	1	2	3	4	5	6	7
F1. Linkages between academia and the workplace							
30. Students on the nursing programme are/should be a resource in the workplace to stimulate the development of nursing	1.07	−0.035	0.030	0.007	−0.020	0.127	0.066
16. Lecturers on the nursing education programme are/should be a resource in the workplace to stimulate the development of nursing	0.380	0.060	−0.026	0.024	−0.076	−0.231	−0.103
F2. Assessment of nursing research and development of the nursing discipline							
04. I think it is interesting to read scientific articles about nursing care	0.042	0.516	−0.033	−0.017	−0.010	−0.043	−0.122
05. (*) The nursing profession does not require research-based knowledge to the same extent as the medical profession	0.031	0.842	−0.097	−0.057	−0.104	0.124	0.037
06. Nursing science and nursing research describes nursing care and makes it visible	0.021	0.543	0.093	0.040	0.053	−0.132	−0.072
07. (*) The nursing profession is a practical profession and does not have to include research	0.019	0.674	0.049	0.149	0.134	0.042	−0.100
10. (*) It is not meaningful to get involved in development work in nursing	0.076	0.389	−0.050	0.096	0.153	−0.065	−0.172
17. (*) Nursing research does not raise the status of the nursing profession	0.022	0.260	−0.088	−0.017	0.154	−0.075	0.093
F3. Language of Research							
21. (*) The language used in nursing research is too complex	−0.061	0.030	−0.956	−0.021	−0.080	0.189	−0.090
09. (*) The language of scientific articles is much too complex for me	0.045	−0.073	−0.697	0.071	0.043	−0.124	0.052
F4. Development of professional and research skills							
01. As a nurse, you must be able to read literature in English	0.104	0.150	−0.184	−0.212	0.052	−0.117	−0.050
03. (*) In the nursing area too much is written and there is too much talk about research and development	−0.059	0.196	−0.064	−0.275	0.159	0.030	0.081
18. A PhD for nurses should be a prerequisite for certain senior positions in nursing	0.022	−0.087	0.007	−0.565	0.008	−0.033	−0.070
22. We should have more nurses in clinical work with a PhD/postgraduate education	0.077	−0.049	0.031	−0.732	−0.022	−0.012	−0.090
24. The results of nursing research must be disseminated better to nurses in their work	0.018	0.121	0.019	−0.232	0.094	−0.188	−0.153
27. Participating in research should be part of the nurse’s job	0.124	0.014	−0.002	−0.427	0.069	−0.335	−0.161
F5. Assessment of nursing research and development as applied in daily professional practice							
15. (*) Nursing research complicates the ordinary work of nursing	0.098	−0.022	−0.206	0.119	0.339	−0.128	−0.032
19. (*) Further training in research and research-based studies is not important for the future	−0.001	0.202	0.079	−0.221	0.449	−0.072	0.031
20. (*) My position as a nurse is sufficiently strong to be able to influence nursing without having knowledge of research	0.018	−0.005	−0.041	−0.145	0.546	0.050	−0.116
23. (*) Taking part in research does not lead to greater professional skill as a nurse	0.189	0.128	−0.008	−0.146	0.300	−0.173	−0.007
26. (*) It is unrealistic to believe one can apply research results to practical nursing.	0.085	0.116	−0.140	−0.071	0.272	−0.083	−0.022
29. (*) I do not bother to find out about research results	0.041	0.084	−0.106	−0.033	0.290	−0.052	−0.131
32. (*) It is not meaningful to devote oneself to research in nursing	−0.088	0.110	−0.049	0.117	0.429	−0.104	−0.391
02. (*) Participating in development work in nursing does not benefit nursing skills	−0.028	0.245	−0.126	−0.164	0.090	−0.011	0.064
08. Research literature on nursing should be available at the workplace (e.g., wards)	0.067	0.377	−0.081	−0.083	−0.102	−0.187	−0.032
F6. Willingness to promote the development of nursing							
11. Being involved in development work in nursing should be part of the nurse’s job	0.034	0.231	0.008	−0.168	−0.030	−0.375	−0.185
12. (*) We do not need nurse scientists to develop patient care, the practise nurses can do that themselves.	0.190	0.176	−0.044	−0.169	0.241	0.251	−0.160
13. I am keen to participate in international scientific conferences	0.076	0.014	−0.048	−0.076	0.155	−0.504	0.000
25. Nursing research is essential for me in my development as a professional nurse	0.103	−0.022	0.004	−0.252	0.165	−0.281	−0.243
F7. Evidence-based practice							
31. It is self-evident that the nursing profession should be based on scientific and reliable experience	0.025	0.160	−0.101	−0.192	−0.035	−0.227	−0.350
33. Nurses should take the time to read research reports	0.020	0.213	−0.121	−0.182	−0.078	−0.087	−0.511
34. Introducing changes and testing new ideas is very important in the nursing profession	0.099	0.013	−0.051	−.050	−0.022	0.000	−0.494
35. I think the questions in this questionnaire are important	0.086	0.009	0.041	−0.105	0.213	0.061	−0.508

(*) reverse items. Extraction method: maximum likelihood. Rotation method: Oblimin with Kaiser normalisation. Rotation converged in 24 iterations. The group of the factors (grey colour).

**Table 4:** Comparison between groups: nurses with diplomas, graduates, and students.

	Nurses with Diplomas *n* = 190		Graduates *n* = 110		Students *n* = 59		ANOVA	
	$\bar{x}$	SD	$\bar{x}$	SD	$\bar{x}$	SD	F	Sig.
F1. Linkages between academia and the workplace	3.83	0.80	4.09	0.68	4.31	0.64	11.316	0.000
F2. Assessment of nursing research and development of the nursing discipline	4.32	0.59	4.38	0.50	4.49	0.37	2.551	0.079
F3. Language of research	3.65	0.89	3.73	0.80	3.85	0.81	1.309	0.271
F4. Development of professional and research skills	3.76	0.60	3.77	0.59	3.87	0.53	0.829	0.437
F5. Assessment of nursing research and development as applied in daily professional practice	4.08	0.52	4.20	0.52	4.37	0.46	7.555	0.001
F6. Willingness to promote the development of nursing	3.98	0.63	4.03	0.62	4.18	0.56	2.420	0.090
F7. Evidence-based practice	4.07	0.57	4.17	0.57	4.43	0.43	9.593	0.000

Mean ($\bar{x}$) and standard deviation (SD), F, Sig. (ANOVA).

## Data Availability

Data are contained within the article.
